# *Sporosarcina pasteurii* can form nanoscale calcium carbonate crystals on cell surface

**DOI:** 10.1371/journal.pone.0210339

**Published:** 2019-01-30

**Authors:** Tanushree Ghosh, Swayamdipta Bhaduri, Carlo Montemagno, Aloke Kumar

**Affiliations:** 1 Department of Mechanical Engineering, University of Alberta, Edmonton, Alberta, Canada; 2 Department of Chemical and Materials Engineering, University of Alberta, Edmonton, Alberta, Canada; 3 Department of Mechanical Engineering, Indian Institute of Science, Bangalore, Karnataka, India; Guangdong Technion Israel Institute of Technology, CHINA

## Abstract

The bacterium *Sporosarcina pasteurii* (SP) is known for its ability to cause the phenomenon of microbially induced calcium carbonate precipitation (MICP). We explored bacterial participation in the initial stages of the MICP process at the cellular length scale under two different growth environments (a) liquid culture (b) MICP in a soft agar (0.5%) column. In the liquid culture, *ex-situ* imaging of the cellular environment indicated that *S*. *pasteurii* was facilitating nucleation of nanoscale crystals of calcium carbonate on bacterial cell surface and its growth via ureolysis. During the same period, the meso-scale environment (bulk medium) was found to have overgrown calcium carbonate crystals. The effect of media components (urea, CaCl_2_), presence of live and dead in the growth medium were explored. The agar column method allows for *in-situ* visualization of the phenomena, and using this platform, we found conclusive evidence of the bacterial cell surface facilitating formation of nanoscale crystals in the microenvironment. Here also the bulk environment or the meso-scale environment was found to possess overgrown calcium carbonate crystals. Extensive elemental analysis using Energy dispersive X-ray spectroscopy (EDS) and X-ray powder diffraction (XRD), confirmed that the crystals to be calcium carbonate, and two different polymorphs (calcite and vaterite) were identified. Active participation of *S*. *pasteurii* cell surface as the site of calcium carbonate precipitation has been shown using EDS elemental mapping with Scanning transmission electron microscopy (STEM) and scanning electron microscopy (SEM).

## Introduction

Biomineralization of calcium carbonate (CaCO_3_) or its precipitation due to chemical alteration of the environment induced by the microbial activity is known as microbiologically induced calcium carbonate precipitation (MICP) [1–4]. Bacteria can participate in the MICP phenomenon by means of various mechanisms such as urea hydrolysis [1, 2], photosynthesis [5, 6], sulfate reduction [7, 8], anaerobic sulfide oxidation [9], biofilm formation [10] and mineral adsorption to extracellular polymeric substances [11, 12]. There has been significant interest in microorganisms that can produce urease (urea amidohydrolase) and hence are able to hydrolyze urea to induce CaCO_3_ precipitation [1, 13–15]. *Sporosarcina pasteurii* (SP), formerly *Bacillus pasteurii*, is a non-pathogenic, endospore forming soil bacterium well known for its ability to precipitate CaCO_3_ through ureolysis [8]. *S*. *pasteurii* has attracted significant attention from researchers for its unique feature of CaCO_3_ precipitation [12, 16–20]. It is being investigated for possible usefulness in a multitude of applications including underground storage of carbon, healing masonry structures of archaeological importance, and long-term sealing of geologic cracks in large-scale structures [3, 15, 20–24].The MICP phenomenon has been exhaustively studied in bulk systems, sand columns [20, 25, 26], and bio-cementation processes [26, 27] to understand the strength provided by the CaCO_3_. The bacterial involvement has also been explored to understand its importance, control mineral deposition rate, urease activity, crystal size and participation of bacterial cells. Hammes and Verstraete [14] reported four very important parameters that affect MICP are pH, dissolved inorganic carbon (DIC), calcium concentration and available nucleation site. The saturation rate of carbonate ion concentration (CO_3_^2−^) is controlled by the first three parameters, and it is believed that bacterial cell surface at the nucleation site can facilitate stable and continuous CaCO_3_ deposition [15]. However, a bacteria-free solution loaded with urease enzyme can also induce CaCO_3_ precipitation. Mitchell and Ferris [17] studied the influence of bacteria on the nucleation of MICP in which a bacteria-free enzyme solution was compared to a bacteria induced environment. The authors reported significant positive effect of bacterial presence (referred as “bacteria-inclusive”) on the increase of size and growth rate of the precipitated crystal although the idea of bacterial control of MICP was rejected. The bacteria-free urease solution showed similarities with the bulk chemical precipitation, while differences were observed in the aqueous micro-environment of bacteria [17].

It has been suggested by other researchers that bacterial cell walls can serve as nucleation sites since bacterial cell surfaces carry negatively charged groups [3, 14, 23, 28–31]. These negative charges can influence the binding of cations (e.g. Ca^2+^) on the cell surface and eventually act as nucleation sites when reacting with carbonate anions from the solution to form insoluble CaCO_3_ [1, 29, 32]. Definitive proofs of involvement of bacterial cell surfaces in complex environments on the nucleation process are rare. Ghashghaei and Emtiazi [33] reported the presence of nanocrystals of CaCO_3_ on cell walls of the bacteria *Enterobacter ludwigii* for experiments performed with a liquid culture. While the data is suggestive of the role of cell wall, a more definite proof is desirable. In a contrasting study, Bundeleva et al. [34] reported on MICP with the anoxygenic *Rhodovulum* sp., where they failed to show the existence of CaCO_3_ on or near live cells. The authors claim the existence of certain cell protection mechanisms against mineral encrustation at the vicinity of live bacteria and they offer the idea of mineral precipitation at a certain distance from the cell surface [34]. In another study, Harris et al. [10] investigated *S*. *pasteurii* biofilm and reported real time biochemical changes during CaCO_3_ precipitation occurred in the proximity of biofilms. They found rapid changes in the pH and Ca^+2^ concentrations near (within 600 μm) the biofilm during the MICP process. The metabolic activity (e.g. ureolysis) in the bacterial micro-environment (proximity of bacterial cells or biofilm) immediately changes the pH and induces CaCO_3_ precipitation. While this study explored the direct participation of live *S*. *pasteurii* biofilm in the MICP process, cellular level contribution by bacterium was not elucidated [10]. In a recent study, Zhang et al. [35] has denied the role of bacterial cell surface or any other negatively charged surface (such as polystyrene microspheres with carboxyl or sulfonic group modified surfaces) in the CaCO_3_ precipitation for their experimental conditions. In contradiction to Zhang’s report, the formation of CaCO_3_ crystals on the negatively charged surfaces were well studied by many researches [36–38]. Negatively charged surfaces terminated with–COOH,–OH, and–NH_2_ functional groups were reported in biomineralization processes of CaCO_3_ by Deng et al. [36]. Authors explored the effect of −OH and −NH_2_ terminated surfaces, where nucleation of amorphous calcium carbonate (ACC) by adsorption of CaCO_3_ clusters from solution was obtained. However, direct formation of calcites was observed on −COOH surface which is more negatively charged. Cao et al. [38] showed similar bio-mineralization on the negatively charged hydrophilic polypeptides at air-liquid interface. The experimental conditions in Zhang’s [35] study were not favorable to facilitate CaCO_3_ precipitation on negatively charged surfaces. Also, the microscopic observations made by Zhang et al. [35] were not detailed enough to capture bacterial cell surfaces at single-cell level. The participation of bacterial cell surface in the MICP process due to ureolytic activity and the proof of CaCO_3_ deposition on bacterial cell surfaces should be reinvestigated. Thus, we see that the issue of the role of bacterial cells on mineral precipitation in MICP remains controversial and detailed studies delineating the exact mechanism leading to the bacterial cell surface deposition of CaCO_3_ in *in-situ* conditions are necessary.

To the address the fundamental mechanism of MICP by *S*. *pasteurii* in complex environments we designed several combinations of MICP compatible liquid medium to confirm direct evidence of CaCO_3_ precipitation on *S*. *pasteurii* cells. Single-cell level electron microscopy and detailed elemental analysis were used to explore initial stages of CaCO_3_ precipitation. We also designed a semisolid-agar column setup, which allows us to directly investigate *in-situ* mineral precipitation. Our setup consists of a 0.5% agar-column that is stab-inoculated and MICP proceeds in the setup as downward traveling conspicuous mineral trails. We investigated the micro and macroenvironment of MICP and conclusively demonstrated the presence of nanometer-sized crystals of CaCO_3_ on the bacterial cell surfaces. While this can be considered as definitive evidence that the cell wall does serve as a nucleation site, other mechanisms of nucleation are not ruled out.

## Materials and methods

### Microorganism and MICP conditions

*Sporosarcina pasteurii* (Miquel) Yoon et al. ATCC 11859 used throughout the MICP study and was cultured in ATCC recommended Tris-YE medium for stock preparation and pilot culture [1]. Live *S*. *pasteurii* cells were collected by centrifugation and washed with saline water. The washing step was repeated until the pH of the supernantant became neutral (pH = 7) and then cells were resuspended in the culture medium. The cell count was maintained at 1×10^6^ CFU/ml of live bacteria approximately. For CaCO_3_ precipitation experiments, *S*. *pasteurii* cultures were prepared in nutrient medium with urea and CaCl_2_ supplements. The liquid CaCO_3_ precipitation medium was prepared with nutrient broth (NB) containing urea (U) and CaCl_2_ (C) and together mentioned as NBUC throughout the study. The NBUC containing the following L^-1^ of double distilled water: 3g nutrient broth (NB); 20g Urea; 2.8g CaCl_2_. Nutrient Broth (NB) was prepared by mixing 5 g peptone; 3 g beef extract and 2 g sodium chloride per 1L of distilled water [39]. The pH of this medium was adjusted to 6.8 ± 0.2. Solution mixtures were sterilized by autoclave wet sterilization method (121°C, 103421 Nm^-2^) for 15 min. Urea and CaCl_2_ solutions were filter-sterilized (PTFE syringe filters, pore size 0.2 μm, Sigma-Aldrich, St. Louis, Missouri, USA) and separately added to the NB medium before bacterial inoculation. All the liquid cultures were incubated in aerobic conditions at 30°C with an orbital shaker operated at 120 rpm. A pH indicator dye (10 μL of phenol red) was added to all the control and test sets after 24 hours of incubation to visualize the pH change. All pH values were measured using pH meter (Mettler Toledo S2 Handheld pH/mV Meter Field Kit) at 25 ^o^C. Peptone, beef extract, urea, NaCl, HCl, NaOH and bacteriological agar were purchased from Fischer Scientific (Thermo-Fisher Scientific, Waltham, Massachusetts, USA). CaCl_2_. 2H_2_O (ACS reagent ≥ 99%) and phenol red solution (#1072420100) were purchased from Sigma-Aldrich (Sigma-Aldrich, St. Louis, Missouri, USA). All the chemicals were used as purchased and solutions were prepared in Milli Q (18.2 MΩ) water.

Different experimental combinations were prepared to observe the role of bacteria and the effect of composition of growth medium on the initiation of CaCO_3_ precipitation in liquid culture. The effect of bacterial metabolic activity (in particular urease activity) and initial pH of growth medium were investigated for various combinations (see Table 1). Experiments were repeated in triplicate, unless otherwise mentioned. NBUC medium with initial pH of 7 and 9 were incubated at 30°C without microbial inoculum and referred to as control set for each combination. The effect of proteins and salts present in the growth medium on the MICP process at two different pH was observed when no microbial inoculum were used. These served as media composition controls. Live *S*. *pasteurii* cells were immediately used as inoculum of NBUC, NBC and NBU media. The dead cells of *S*. *pasteurii* were prepared by heat killing of bacterial cells, autoclaving them at 121°C for 20 min. Heat killed dead *S*. *pasteurii* cells were collected and washed with saline medium before addition to NBUC media. Table 1 shows all the setup where live and dead *S*. *pasteurii* are mentioned in subscript to the media composition.

**Table 1:** Experimental setups implemented for liquid culture studies.

Experimental sample (n = 3)	NB medium components and initial pH			
	Urea	Calcium chloride	SP (Live/Dead)	Initial pH
NBUC-7	+	+	-	7
NBUC-9	+	+	-	9
NBUC-7SP_dead_	+	+	+ Dead	7
NBUC-7SP_live_	+	+	+ Live	7
NBC-7SP_live_	-	+	+ Live	7
NBU-7SP_live_	+	-	+ Live	7
NBUC-9SP_dead_	+	+	+ Dead	9
NBUC-9SP_live_	+	+	+ Live	9
NBUC-7 Urease	+	+	-	7

‘+’ or ‘-’ represents addition or deletion of components to the medium

SP stands for *Sporosarcina pasteurii*

SP_dead_ or SP_live_ represents the addition of killed or live bacterial inoculums to NBUC medium

NBC- only CaCl_2_ supplemented NB medium

NBU-only urea supplemented NB medium

### MICP in semisolid-agar medium

Visualization of MICP was finally aided by semisolid-agar columns, prepared by following Bang’s urea-CaCl_2_ liquid medium [1] with modification. The modified media contain (w/v) peptone 0.15%; beef extract 0.09%, sodium chloride 0.06% and CaCl_2_, 0.28%. The pH of the medium was adjusted to 7.0 and then 0.5% agar was added prior to autoclaving. Urea (2%) was added separately after autoclaving when media temperature cooled down to approximately 50–60°C. To create the agar columns, 10 ml of liquid agar was poured into upright test tubes and allowed to cool inside a biosafety cabinet, which finally resulted in columns of approximately 5 cm in length. Subsequently, these agar columns were inoculated by stabbing the free surface of the agar column with pre-cultured *S*. *pasteurii* using a stabbing needle. Fresh liquid media were poured on to the agar column to prevent drying of the agar surface. The bacteria inoculated columns along with control sets were incubated at 30°C for a maximum duration of 7 days.

### Electron microscopy

Bacterial broth cultures grown in different culture medium (experimental combinations from Table 1) were observed for CaCO3 deposition using field emmission scanning electron microscopy with EDS (Zeiss Sigma FESEM, Oberkochen Germany) and High resolution transmission elelctron microscopy (JEOL JEM-ARM200CF S/TEM with EDX, Massachusetts, US). For all the experimental setup with initial pH 7, culture aliquots were obtained after 24 hours of incubation at 30°C. The setups with the initial pH 9, bacterial cells were collected within 1 hour of inoculation. Control sets were treated accordingly. The SEM and TEM samples were prepared by fixing and dehydration in graded ethanol upto 100% ethanol[40, 41]. Agar columns were also investigated using both SEM and TEM. The cross-sections of the agar-column were fixed and dehydrated for SEM imaging. Every SEM sample was gold sputtered (Denton Vacuum, Desk II, Moorestown, New Jersey) before imaging.

Prior to TEM, NBUC agar columns were cut at approximately mid-height (~ 2.5 cm) where bacterial activity were observed by optical microscopy. Agar slices were cut into small cubes and fixed in solution containing 2.5% glutaraldehyde, 2% paraformaldehyde in 0.1 M phosphate buffer (pH = 7.4) for 30 min. The fixation process allows *in-situ* preservation of *S*. *pasteurii* cells and its micro-environment. Post-fixation treatment of 1% Osmium tetroxide in 0.1 phosphate buffer was performed for 1 hour. Standard protocols for buffer washing and dehydration through graded alcohol were followed [42]. Samples were infiltrated with Spurr’s resin (1:1 of Ethanol: Spurr mixture) for 3 hours and then kept in 100% Spurr for 24 hours. Samples were then embedded in flat molds with fresh Spurr’s resin and cured overnight at 70°C. Cured embedded resin capsules were sectioned in 70–90 nm thin sections using ultramicrotome (Reichert-Jung UltraCut E, Vienna, Austria) and mounted on copper grid for transmission electron microscopy (TEM, Philip-FEI, Morgangni 268, Oregon, USA) operated at 80kV.

### X-ray diffraction spectroscopy (XRD) and energy dispersive spectroscopy (EDS)

Liquid medium and agar column samples were analysed for consolidated mineral precipitation. For XRD analysis, CaCO3 precipitate from agar column were collected, air dried and ground to powder. Ssamples were scanned by X-ray diffractometer (powder XRD, Rigaku Ultimate IV, Rigaku Corporation, Tokyo, Japan) and analysed for chemical components (Jade XRD pattern analysis software). EDS were performed alongwith SEM and TEM imaging on the bacterial cell surface and on to the surface of precipitated mineral. Chemical components were determined using EDS elemental mapping.

## Results and discussion

### The MICP process

**Fig 1:**
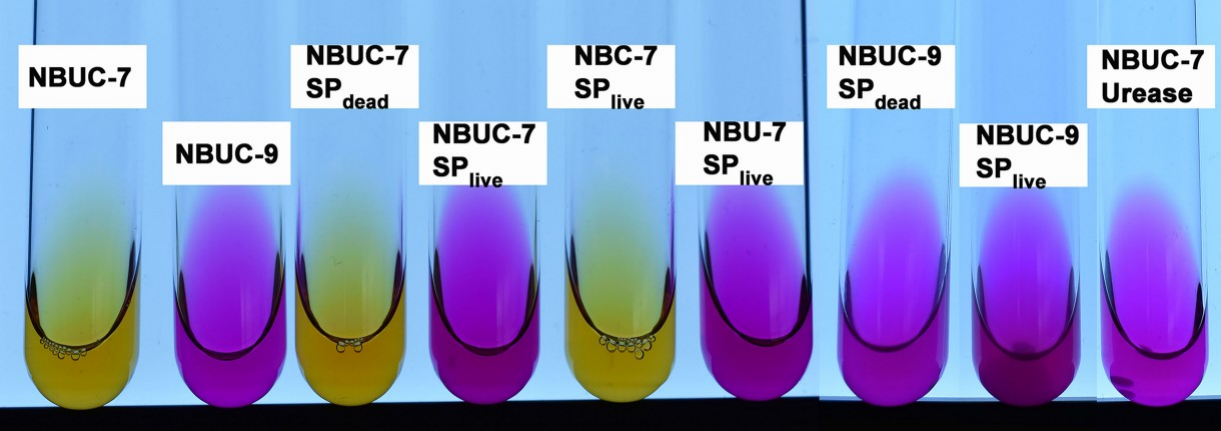
Parametric evaluation of the MICP phenomenon by *S*. *pasteurii* in liquid media. Phenol red was used as pH indicator dye in culture tubes showing the final pH after 24h of culture. The text indicates the initial culture condition (see also Table 1).

Fig 1 shows the parametric evaluation of the MICP phenomenon for nine different setups after 24 hours of culture. Phenol red (pH indicator dye) was added to all the nine cases to evaluate change in pH from neutral to alkaline (yellow/orange to pink). NBUC-7, NBUC-9 and NBUC-7Urease denote the control sets, which were not inoculated with *S*. *pasteurii* (refer to Table 1). For sets NBUC-7SP_live_, NBU-7SP_live_ and NBUC-7Urease the change of color indicates ureolytic activity. A comparison of NBUC-7SP_dead_ and NBUC-7SP_live_, suggests that bacterial activity led to change in pH; precipitation was also observed for NBUC-7SP_live_. In the control set NBC-7SP_live_ (without urea), bacterial activity was unable to change medium pH and also no precipitation was observed even in the presence of same molar concentration of Ca^+2^ as NBUC-7SP_live_. Hence NBC medium served as calcium positive control, where media conponent unable to induce precipitation. On the other hand, NBU-7SP_live_ served as calcium negative control and did not show any precipitation due to absence of Ca^+2^, despite a change in pH.

The biophysical phenomenon of MICP has been investigated by several researchers and the following sequence of reactions (Eqs 1–6) has typically been suggested to lead to MICP [23, 26, 43]:

$$CO(NH_{2}{)}_{2}+H_{2}O\overset{ureolysis}{\to }NH_{2}COOH+{NH}_{3}$$ (1)

$$NH_{2}COOH+H_{2}O\to {NH}_{3}+H_{2}CO_{3}$$ (2)

$${{2NH}_{3}+2H}_{2}O\to 2NH_{4}^{+}+2OH^{-}$$ (3)

$$2OH^{-}+H_{2}CO_{3}\to CO_{3}^{2-}+2H_{2}O$$ (4)

$$Cell+Ca^{2+}\to Cell∼Ca^{2+}$$ (5)

$$Cell∼Ca^{2+}+CO_{3}^{2-}\to Cell∼CaCO_{3}$$ (6)

**Fig 2:**
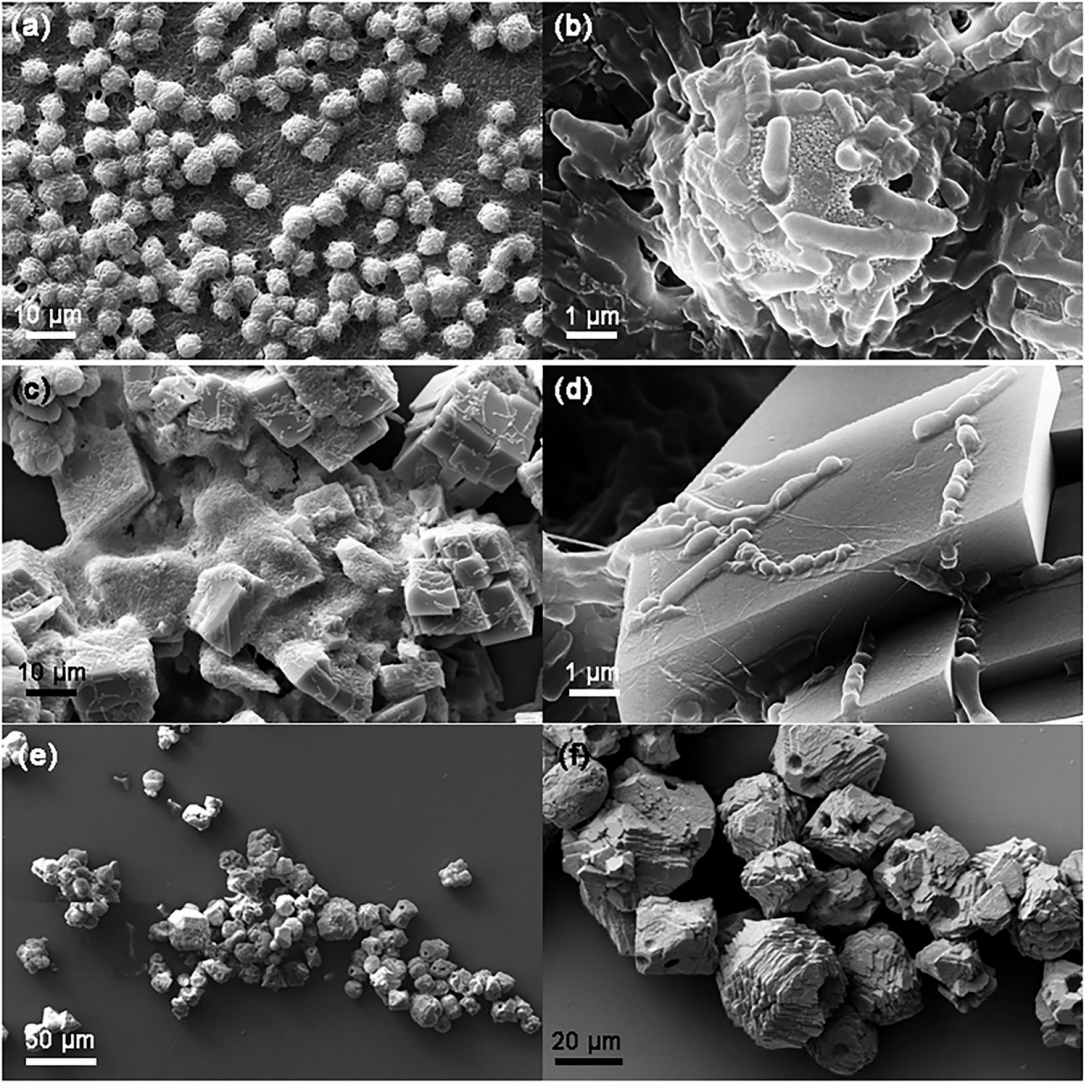
SEM images showing the effects of culture conditions on precipitate morphology. (a) Effect of initial pH of NBUC medium (NBUC-9SP_live_) showing bacteria bound to crystalline microspheres. (b) Zoomed in section of (a). (c) Typical calcite precipitation in NBUC-9SP_dead_ medium with dead *S*. *pasteurii* cells. The zoomed in image (d) showing orthorhombic crystal structure of precipitates (e) precipitation induced by Urease (EC 3.5.1.5) supplemented condition (NBUC-7 Urease) where bacteria were absent. (f) zoomed in section of (e).

The precipitates obtained from NBUC-9SP_live_, NBUC-9SP_dead_ and NBUC-7Urease was imaged using FESEM and the results are shown in Fig 2. Different morphologies of crystalline precipitates were observed in the different cases. For conditions of Fig 2A to 2D, precipitation within a very short time-scale (~ few minutes) was observed. For the NBUC-9SP_dead_ set, rhombohedral crystalline precipiates were observed (Fig 2C and 2D). The morphology of precipitates for the NBUC-9SP_live_ set (Fig 2A and 2B), was distinctly different from the NBUC-9SP_dead_ set (Fig 2C and 2D). In Fig 2A and 2B, precipitation in the form of microspheres with a significant population of embedded bacterial cells was observed. These results suggest that prcipitation in the NBUC-9SP_dead_ was chemical in nature, whereas NBUC-9SP_live_ set precipitation is likely both chemically and biochemically induced. The change in medium pH due to urease activity was supported by the urease enzyme supplemented (NBUC-7urease) test (Fig 2E and 2F). The MICP process is only observed for the NBUC-7SP_live_ and NBUC-9SP_live_ sets (see also Figure A in S1 File).

### Nanoscale environment of *S*. *pasteurii*

**Fig 3:**
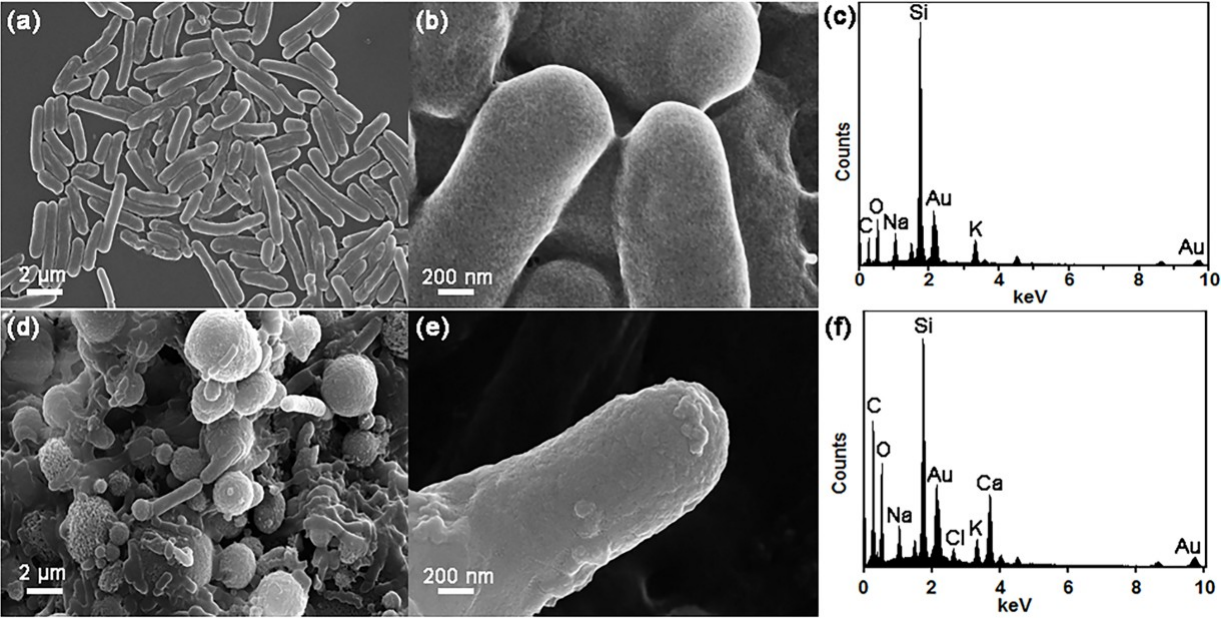
SEM and EDS analysis of *S*. *pasteurii*. (a-b) SEM images of *S*. *pasteurii* control cells from NBC-7SP_live_ showing bacterial cells with no evidence of crystalline deposits on the cell wall. (c) EDS spectra collected on (b). (d-e) SEM images of *S*. *pasteurii* cells from NBUC-7SP_live_ clearly showing presence of crystalline deposits on the cell-wall. (f) EDS spectra collected on (e) and clearly showing presence of Ca.

To investigate the nanoscale environment of the *S*. *pasteurii* cell surface, FESEM imaging along with EDS analysis were performed on the *S*. *pasteurii* cell surface at the single-cell level. Fig 3 depicts the data that was obtained for the NBC-7SP_live_ (Fig 3A to 3C) and NBUC-7SP_live_ (Fig 3D and 3F). Fig 3A and 3B shows no evidence of any precipitation on the cell surface, whereas for the NBUC-7SP_live_ set definitive precipitation can be observed on the cell surface. Further, EDS elemental map spectrum were obtained (at 20kV, dwelling time 50μs, process time 5s) for *S*. *pasteurii* cell surface from NBC-7SP_live_ (Fig 3C) and NBUC-7SP_live_ (Fig 3F) and they confirmed the presence of Ca^+2^ only on *S*. *pasteurii* cell from NBUC-7SP_live_ set. Note, that the NBC-7SP_live_ setup gives us an positive control where Ca^+2^ ions were present in same concentration (25 mM CaCl_2_) as in NBUC-7SP_live_ set. For the NBUC-9SP_dead_ set, no traces of cell surface deposition were found on the dead *S*. *pasteurii* cell. Fig 3E shows *S*. *pasteurii* cell surfaces were observed to have islands of nano-sized deposition with strong intensities of Ca Kα. It provides strong support for the hypothesis that bacterial cell surfaces can serve as nucleation sites for CaCO_3_ precipitation or the MICP phenomenon.

The precipitation found in NBUC-7SPlive were mostly spherical, nano to micron sized and located on or adjacent to *S*. *pasteurii* cells. A plausible mechanism is schematically represented (Figure B in S1 File) and compared with FESEM images to relate all the observations. *S*. *pasteurii* cells were observed to covered by CaCO_3_ either partially or fully. Spherical growth of CaCO_3_ was also observed in aggregation with *S*. *pasteurii* cells. The partially covered (partially encrusted) *S*. *pasteurii* cells were found to have islands of CaCO_3_ on cell surface. Whereas, fully encrusted cells found to have terminal spherical growth of CaCO_3_. The over-growth of nano/micron-sized CaCO_3_ may result in the death of fully encrusted bacteria leaving the impression of its existence. The structure of calcium carbonate precipitate may differ depending on the media constituents (chemical content), pH, protein (enzyme content) and polymeric substances (EPS content) and other medium additives. Thus, the morphology of the crystals can provide important clues about the precipitation pathway. Figs 2 and 3 also suggests that the MICP phenomenon can be segregated into two spatial domains (i) the nanoscale neighborhood of the cell surface (ii) the bulk medium, which can be called the meso-scale environment of the cell. Our study was aimed at understanding the relevance of the former.

**Fig 4:**
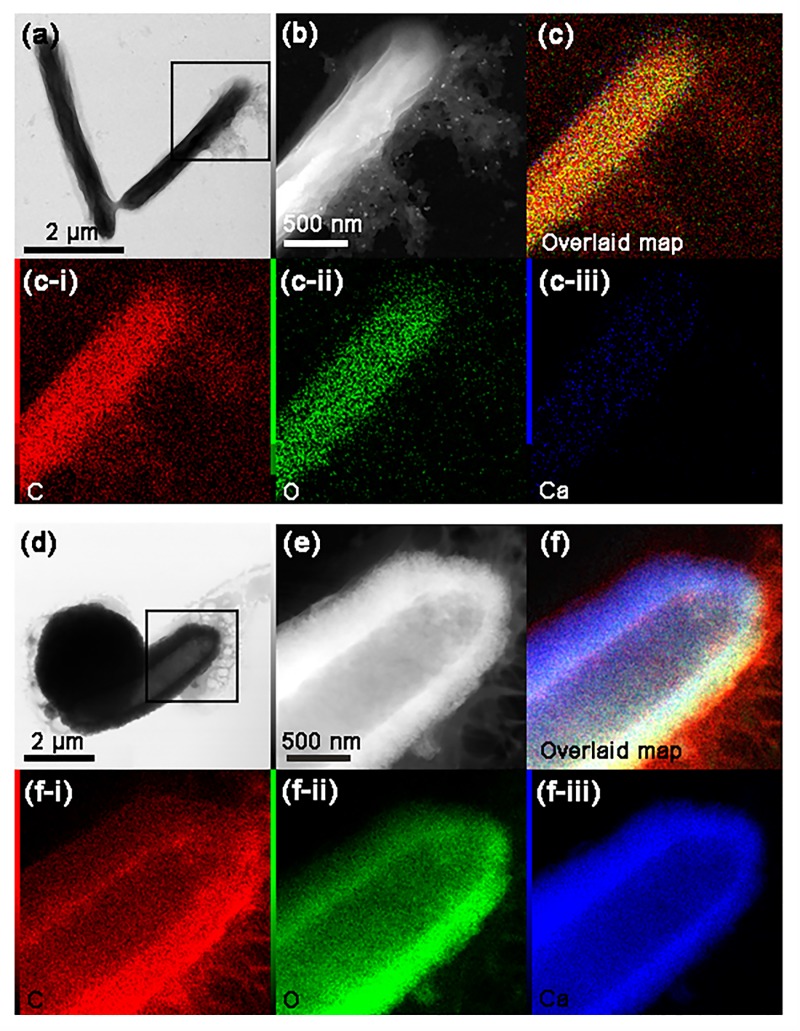
STEM-BF and DF images and EDS elemental mapping on single *S*. *pasteurii cells*. (a) STEM-BF image of *S*. *pasteurii* cells from NBC-7SP_live._ (b) zoomed in STEM-DF image at boxed site (c) A cumulative map created by overlaying EDS elemental mapping of (b). c(i-iii) show individual elemental distribution map for C, O and Ca along the *S*. *pasteurii* cell surface. (d) STEM-BF image of *S*. *pasteurii* cell from NBUC-7SP_live._ (e) zoomed in STEM-DF image at boxed site (f) cumulative overlaid map f(i-iii) show individual elemental distribution map for C, O and Ca along the *S*. *pasteurii* cell surface.

**Fig 5:**
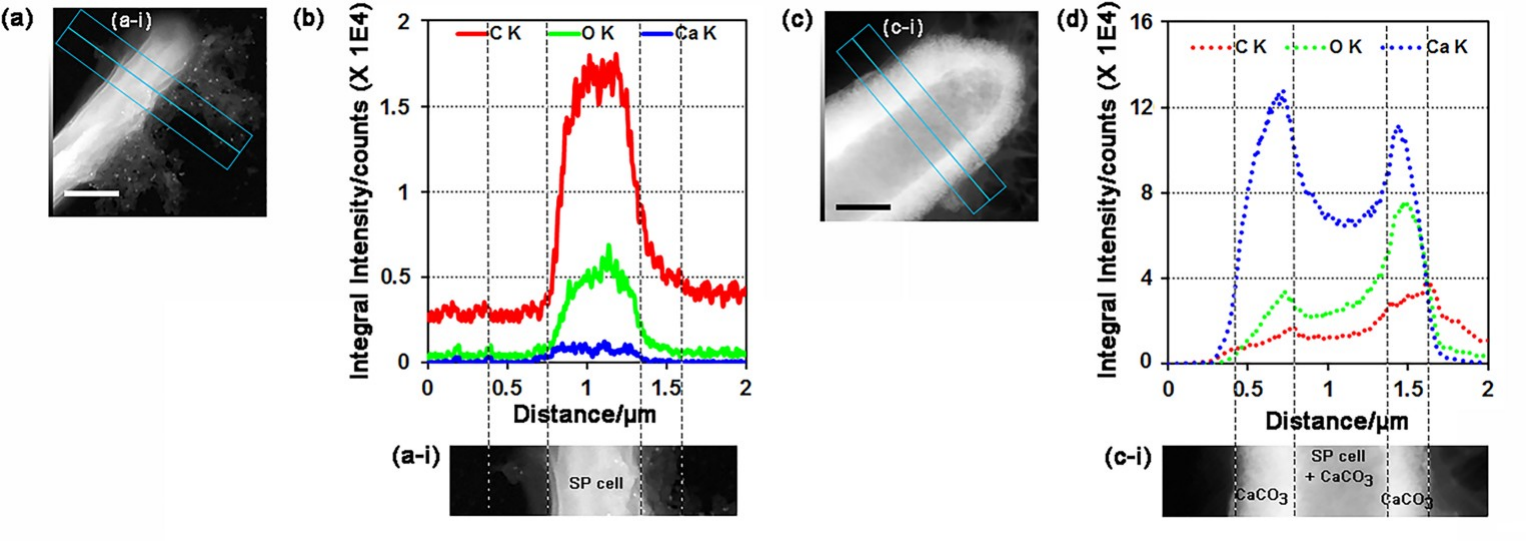
STEM and extracted line profile of CaCO_3_ deposited on *S*. *pasteurii* cell surface. The dark field TEM images representing the control *S*. *pasteurii* cell from NBC medium (a) and from NBUC medium (c). Scale bar is 500 nm. The area demarcated by the blue rectanges is the area where corresponding element intensities were integrated and presented along the middle blue line for a distance of 2 μm. The (b and d) EDS line scan showing the extracted intensity profile for K series of C, O and Ca. Solid lines (b) showing C, O and Ca intensities obtained from NBC-7SP_live_ cell surface where dashed line (d) intensiy profile for NBUC-7SP_live_ cell surface.

The *S*. *pasteurii* cell surface was further investigated by STEM associated with EDS (Fig 4). Fig 4A and 4B show STEM images of a single *S*. *pasteurii* cell obtained from NBC-7SP_live_ (Fig 4A ans 4B) and NBUC-7SP_live_ (Fig 4D and 4E). EDS elemental mapping was obtained for both samples. A cumulative map image was created for single-cell surface from NBC-7SP_live_ (Fig 4C) and NBUC-7SP_live_ (Fig 4F) to demonstrate co-localization of elements corresponding to EDS response. The distribution of individual elements of CaCO_3_ (C, O and Ca) were imaged separately for NBC-7SP_live_ (Fig 4Ci to 4Ciii) and NBUC-7SP_live_ (Fig 4Fi to 4Fiii). Fig 4 clearly shows the cell sample from NBUC-7SP_live_ encrused with calcium on the periphery of the cell surface indicating the nucleation of CaCO_3_. The map spectrum was quantified by using AZTech-EDS software and elemental signals were integrated along the *S*. *pasteurii* cell surface for NBC-7SP_live_ (Figs 5A and 4B) and NBUC-7SP_live_ (Figs 5C and 4D). The cross-sectional line profiles (Fig 5B and 5D) corresponding to image sections (Fig 5A-i and 5C-i) showed the intensities quantified for C, O and Ca. The line profile for Fig 5A-i is shown in Fig 5B and 5A very weak traces of Ca intensity on the cell boundary can be seen. On the other hand, image section of NBUC-7SP_live_ (Fig 5C-i) shows a strong signal from Ca along the cell boundary (Fig 5D) resulting in to two Ca-peaks. Remarkable increase in intensities for Ca at the fully encrusted cell towards both edges (Fig 5C-i, CaCO_3_ regions) was observed, which indicates a cell surface coating of Ca. Similar increase in intensities along the *S*. *pasteurii* cell boundary were also found for C and O which confirms the formation of CaCO_3_ coating on *S*. *pasteurii* cell. The Ca content on *S*. *pasteurii* cells in NBUC medium was found to be ~600 fold higher along the bacterial cell than obtained in the NBC-SP cells. The Ca content on the NBC-SP cells were negligible compared to NBUC-SP cells, which rule out the adsorbtion of Ca^+2^ on the bacterial extrapolymeric substances or to the medium components when ureolytic activity is absent.This gives us a direct evidance of *S*. *pasteurii* cell surface deposition The elemental content were also quantified and compared for C, O and Ca from the intensities obtained from mapping EDS spectrum (Figure C in S1 File).

### Agar column study

**Fig 6:**
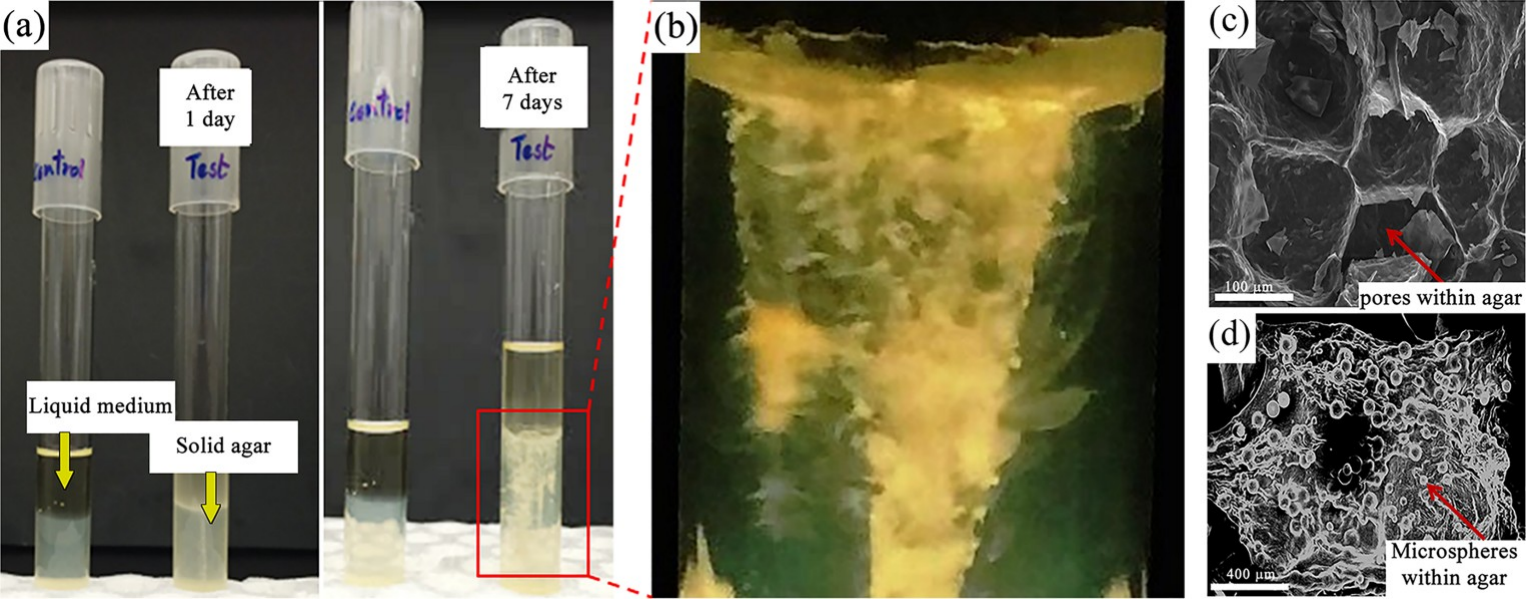
MICP in agar column (a) Two samples containing the semi-solid 0.5% agar medium in two identical 20 mL test tubes. The one to the left is the control sample which is devoid of any bacterial cells. To the right is the *S*. *pasteurii* inoculated sample where the resulting mineral precipitation has left a conspicuous trail. Images were taken after 1 day and 7 days of the inoculation. (b) Blown out magnified image of the mineral deposition. (c-d) SEM image of agar showing pores in control set (c) and numerous mineral microspheres after 7 days (d) of incubation.

To understand growth of *S*. *pasteurii* in a less-mobilized and porous environment, an agar column with stab culture was observed for a period of one week. The agar-cloumn samples were fixed with glutaraldehyde allowing us to study the MICP phenomenon *in-situ*. Fig 6A shows the original culture tubes containg growth spans after day-1 and day-7. Extensive calcium carbonate deposition can be prominently seen in Fig 6B after 7 days of incubation. The agar media acted as soft, porous and transperant nutrient riched environment to monitor the bacterial motility and calcite precipitation. Fig 6C depicts a scanning electron microscopy image of the agar column, where porous structures can clearly be seen. Fig 6D also shows the presence of deposits in the agar column after 7 days of bacterial activity. *S*. *pasteurii* bacterium posses flagellum, which allows it to navigate the porous structure of the agar column.

**Fig 7:**
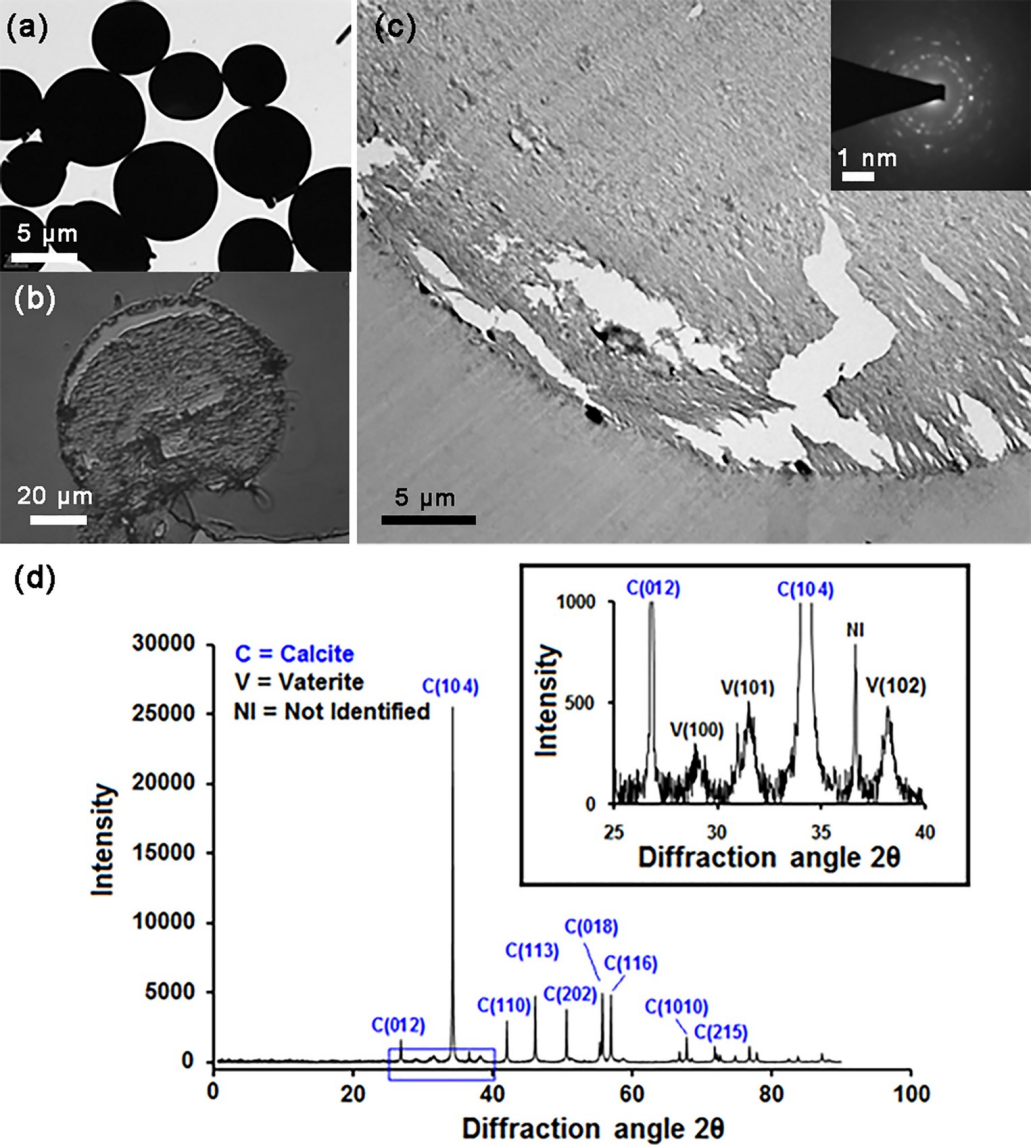
Optical and electron microscopy images of semi-solid agar grown CaCO_3_ microspheres and its XRD pattern. (a) TEM images of CaCO_3_ microspheres deposited far from SP cells (macro-environment) (b) An optical microscopy image and an ultrathin (80 nm) section of agar column with CaCO_3_ microspheres, stained with crystal violet. (c) TEM image of a portion of the ultrathin (80 nm) section. (inset) The SAED pattern of the crystalline structure. (d) XRD plot indicates the formation of calcite and vaterite polymorphs. (inset) clearly demonstrates presence of vaterite.

The primary advantage of the agar-column experimental setup is *in-situ* visualization and easy access for supporting characterizations. The agar column was section at a depth of 2.5 cm after a period of 7 days and crystalline depositions were characterized by other forms of microscopy. Specifically, the agar colunm was characterized using optical microsopy, transmission electron microscopy (TEM), selected area diffraction pattern (SAED) and elemental characteization was determined by powder XRD and energy-dispersive X-ray spectroscopy (EDS). Optical microscopy of ultrathin (~80 nm) sections of the agar medium revealed depositions of crystalline mircrospheres profusely within the agar column. Optical and TEM imaging revealed that the visible white deposits within the column were mostly crystalline micropheres of about 10–50 μm in diameter (Fig 7A).

**Fig 8:**
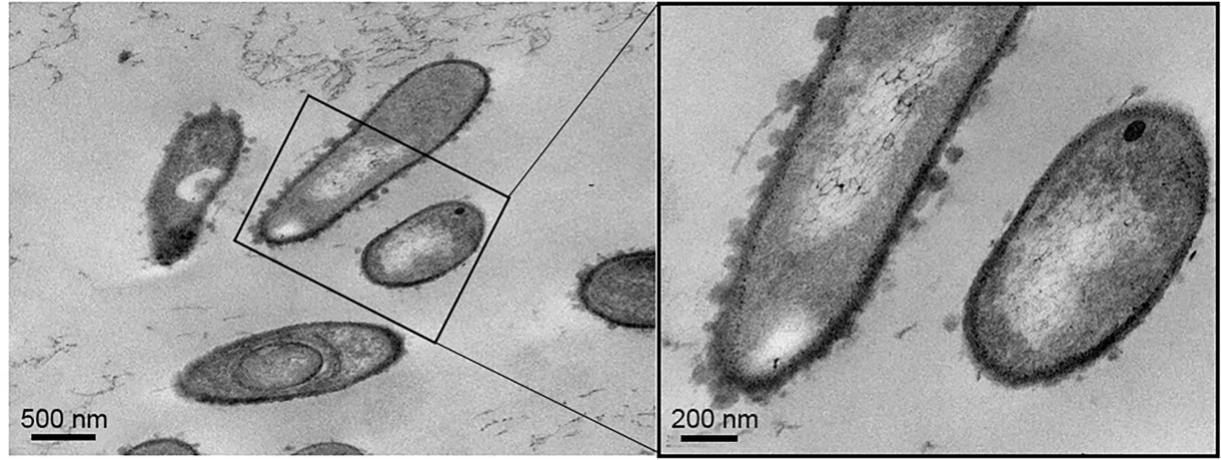
TEM images of ultrathin sections of the agar medium depicting the presence of *S*. *pasteurii* cells with cell surface depositions of nanometer sized CaCO_3_.

TEM images of the microspheres (Fig 7B) shows a crystal violet stained corss-section of microsphere. Fig 7C shows the TEM image of the same with a SAED pattern (Inset of Fig 7C) which proves the crystalline nature of the precipitates and also consistent with the Miller indices of calcium carbonate observed in XRD. Fig 7D shows the powder XRD result of the collected precipitate used to identify the crystalline phases of the inorganic compounds. The identified signature peaks of calcite (Fig 7D) at 2θ valus of 26.83^o^, 34.26^o^ and 42.01^o^ respectively corelated with lattice (hkl) indiced of (012), (104) and (110). Low intensity vaterite peaks (Fig 7D Inset) were also identified at 2θ values of 28.98^o^, 31.52^o^ and 38.22^o^ corelated with lattice (hkl) indiced of (100), (101) and (102), respectively. TEM imaging of the micro-environment of bacterial cell surface shed light on the likely nucleation route for the calcite microspheres. The 80 nm ultra-thin sections of agar column containing embedded bacteria shows in Fig 8 clearly depict the nanoscale environment of the bacterium cell surface. Magnified images indicate nanoscale spherical depositions on cell surface (black rectangle) and needle-like depositions in the surrounding agar media. These results are definitive proof that SP can participate in the nucleation of crystals via its cell surface. However, we would like to reiterate that other nucleation mechanisms are not ruled out by our study.

It is believed that the MICP process is driven primarly by urea hydrolysis [1, 10, 14, 17, 25, 35]. Caclium carbonate precipitation and the crystal morphology are likely influenced by two major factors: i) biochemical induction and/or ii) purely chemical induction. A process driven by biophysical/biochemical induction can be expected to be significantly slower than a purely chemical induction driven process. Culture media which were initially alkaline, resulted in a comparatively faster precipitation and hence were sampled after one hour; media with an initially neutral pH resulted in a slower precipitation and hence were sampled after 24 hours. This difference in time-scales suggests that the former were chemically induced while the latter were biochemically induced. In case of biochemical induction, the process is likely to be extremely complex and we found that the nanoscale environment of the cell played a crucial role in the MICP process. Bacterial cell walls are expected to possess negatively charged units [3, 14, 23, 29], which likely resulted in a transport of Ca^2+^ ions towards the cell surface facilitating nucleation at the cell surface. Additionally, urea hydrolysis by the bacterial cell may have resulted in a highly alkaline (rich in OH^-^ ions) domain around the cell surface creating the “nanoscale neighborhood” which eventually served as sites of CaCO_3_ precipitation. Also, the possibilities of the presence of highly proteinaceous S-layer [29] or extra-polymeric substances (EPS)[10] in the nucleation process can not be discarded. Though the participation of bacterial cell surface in the CaCO_3_ precipitation is definite, the particular reason(s) behind still require more research.

## Conclusions

In this work, we explored the MICP process by the bacterium *Sporosarcina pasteurii*. The participation and behavior of *S*. *pasteurii* cells at different pH of the medium as well as at different medium composition was investigated. Ureolytic *S*. *pasteurii* cells were found to possess cell surface CaCO_3_ and it was prominent deposited on the cell surface. To explore *in situ*, semi-solid agar (0.5%) was used to create a porous column, which was inoculated with a stab culture. As *S*. *pasteurii* cells migrated down the agar column they left conspicuous trail of crystals. Samples of the agar column at different locations were taken and subjected to microscopy and we found that the crystal train consisted of calcite microspheres, which on closer inspection were found to be an aggregate of needle-like nanoscale crystals. Moreover, cells whose surface contained calcite nanocrystals were also observed confirming the hypothesis that cell surface plays a role in nucleation.

## Supporting information

S1 File (PDF)

S2 File (XLSX)

S3 File (XLSX)

S4 File (XLSX)
