# Supplementary material for: Sporosarcina pasteurii can form nanoscale calcium carbonate crystals on cell surface
Source: PLoS One. 2019 Jan 30;14(1):e0210339. doi: 10.1371/journal.pone.0210339 (PMC6353136; doi:10.1371/journal.pone.0210339)
Supplement: S1 File — (PDF) [file pone.0210339.s001.pdf]

Supplementary Information for

Sporosarcina pasteurii can form nanoscale calcium carbonate crystals on cell surface

Tanushree Ghosh[1], Swayamdipta Bhaduri[1], Carlo Montemagno[2†] and Aloke Kumar[3]*

[1]Department of Mechanical Engineering, University of Alberta, Edmonton Alberta, Canada

[2]Department of Chemical and Materials Engineering, University of Alberta, Edmonton Alberta, Canada

[3]Department of Mechanical Engineering, Indian Institute of Science, Bangalore Karnataka, India

*__Corresponding author__

E-mail: alokekumar@iisc.ac.in(AK)

†Decreased

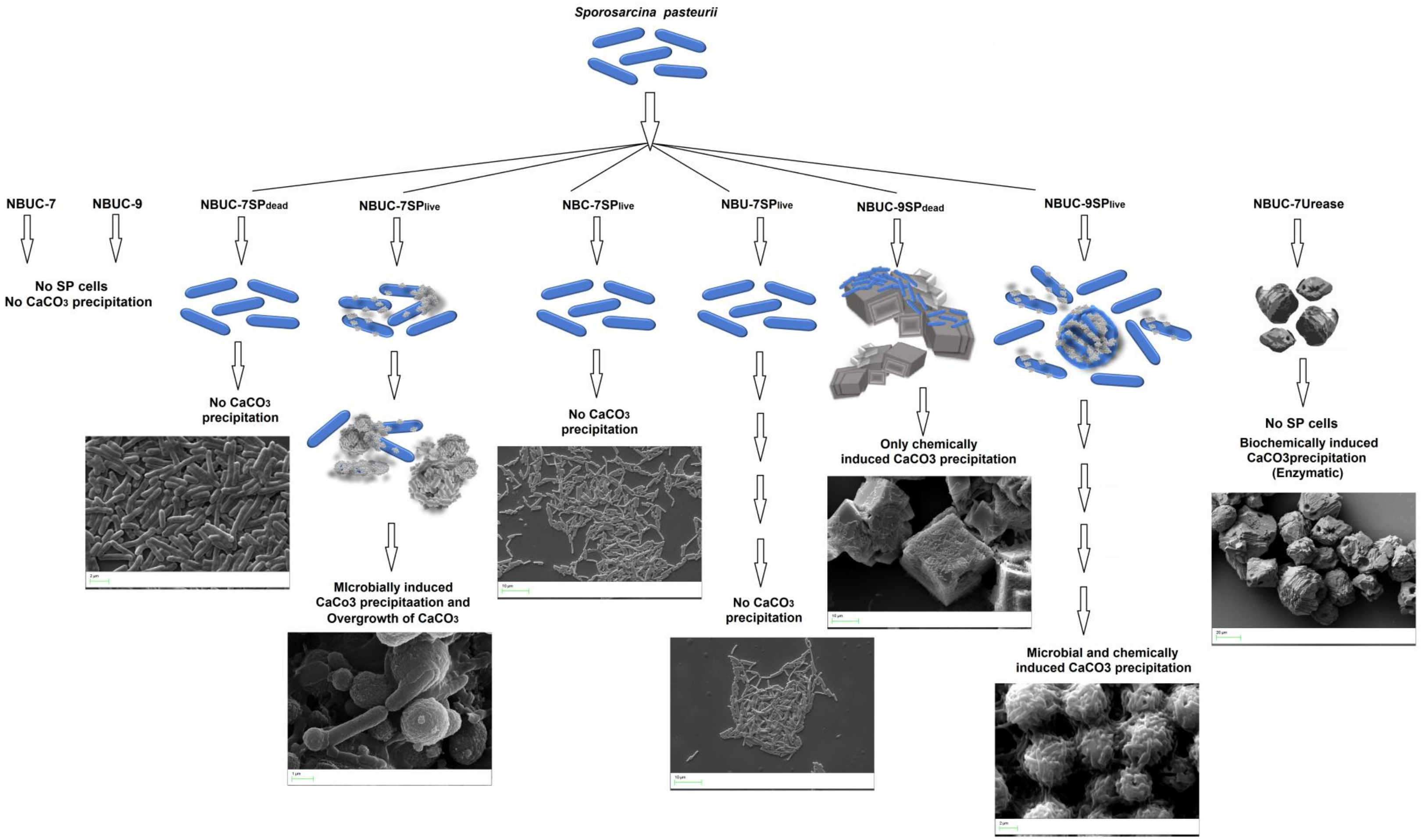

Figure A. Schematic representation of the various observations of precipitation in liquid media. The text indicates the initial culture condition. (see also Table-1). All the possible outcomes were compared with FESEM images for each set.

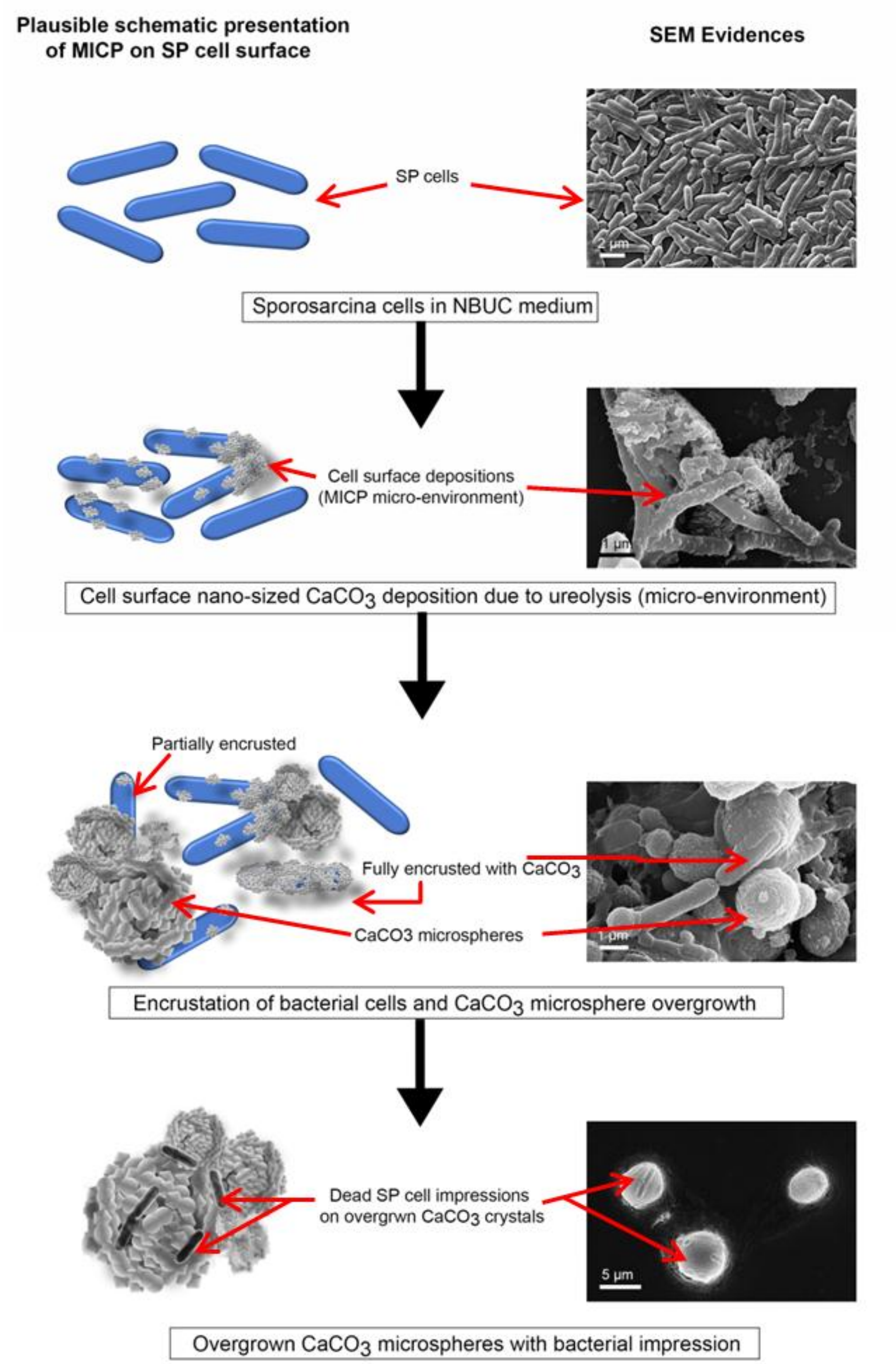

Figure B. Schematic representation of plausible mechanism of *S. pasteurii* cell participation in MICP process, compared with evidences observed in FESEM images for NBUC-7SP_{live}.

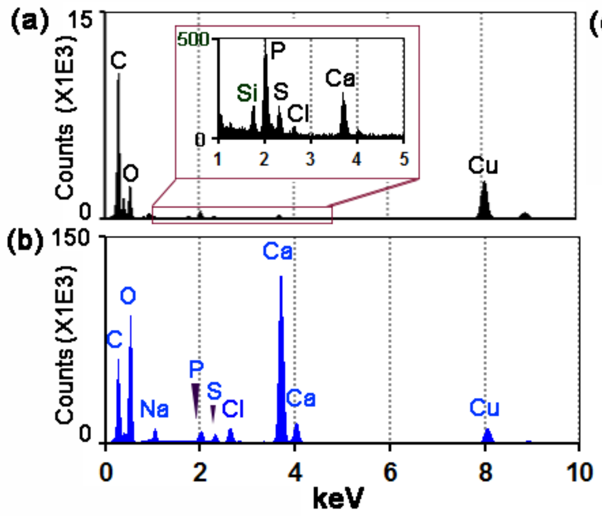

(c) Elemental quantification

Elements (NBC-7SPlive)	Mass %	Atomic %
C	82.6	88
O	12.7	10.2
Ca	0.01	0
**Elements (NBUC-7SPlive)**	**Mass %**	**Atomic %**
C	11.3	24.4
O	17.5	28.5
Ca	49.4	32.1

Figure C. EDS spectrum obtained from *S. pasteurii* cells from NBC medium (a) and from NBUC medium (b). The Cu intensities for both the spectrum are attributed to the Cu-grid for TEM sample mount. The elemental quantification table (c) showing Mass % and Atomic % of corresponding elements.
